# Vasoconstriction Response to Mental Stress in Sickle Cell Disease: The Role of the Cardiac and Vascular Baroreflexes

**DOI:** 10.3389/fphys.2021.698209

**Published:** 2021-11-04

**Authors:** Wanwara Thuptimdang, Payal Shah, Maha Khaleel, John Sunwoo, Saranya Veluswamy, Roberta M. Kato, Thomas D. Coates, Michael C. K. Khoo

**Affiliations:** ^1^Department of Biomedical Engineering, University of Southern California, Los Angeles, CA, United States; ^2^Hematology Section, Children’s Center for Cancer, Blood Disease and Bone Marrow Transplantation, Children’s Hospital Los Angeles, University of Southern California Keck School of Medicine, Los Angeles, CA, United States; ^3^Division of Pulmonology, Children’s Hospital Los Angeles, University of Southern California Keck School of Medicine, Los Angeles, CA, United States

**Keywords:** sickle cell anemia, mental stress, vasoconstriction, baroreflex, autonomic nervous system

## Abstract

Recent studies have shown that individuals with sickle cell disease (SCD) exhibit greater vasoconstriction responses to physical autonomic stressors, such as heat pain and cold pain than normal individuals, but this is not the case for mental stress (MTS). We sought to determine whether this anomalous finding for MTS is related to inter-group differences in baseline cardiac and vascular autonomic function. Fifteen subjects with SCD and 15 healthy volunteers participated in three MTS tasks: N-back, Stroop, and pain anticipation (PA). R–R interval (RRI), arterial blood pressure and finger photoplethysmogram (PPG) were continuously monitored before and during these MTS tasks. The magnitude of vasoconstriction was quantified using change in PPG amplitude (PPGa) from the baseline period. To represent basal autonomic function, we assessed both cardiac and vascular arms of the baroreflex during the baseline period. Cardiac baroreflex sensitivity (BRSc) was estimated by applying both the “sequence” and “spectral” techniques to beat-to-beat measurements of systolic blood pressure and RRIs. The vascular baroreflex sensitivity (BRSv) was quantified using the same approaches, modified for application to beat-to-beat diastolic blood pressure and PPGa measurements. Baseline BRSc was not different between SCD and non-SCD subjects, was not correlated with BRSv, and was not associated with the vasoconstriction responses to MTS tasks. BRSv in both groups was correlated with mean PPGa, and since both baseline PPGa and BRSv were lower in SCD, these results suggested that the SCD subjects were in a basal state of higher sympathetically mediated vascular tone. In both groups, baseline BRSv was positively correlated with the vasoconstriction responses to N-back, Stroop, and PA. After adjusting for differences in BRSv within and between groups, we found no difference in the vasoconstriction responses to all three mental tasks between SCD and non-SCD subjects. The implications of these findings are significant in subjects with SCD since vasoconstriction reduces microvascular flow and prolongs capillary transit time, increasing the likelihood for vaso-occlusive crisis (VOC) to be triggered by exposure to stressful events.

## Introduction

Sickle cell disease (SCD) is caused by a single mutation in the β-globin gene, resulting in the production of sickle hemoglobin which polymerizes after deoxygenation (Rees et al., 2010). The polymerization changes liquid hemoglobin into a solid, transforming the flexible RBC into sickled-shaped RBC that tend to obstruct microvascular flow. The clinical manifestation of extensive obstruction of microvascular flow is episodic painful vaso-occlusive crisis (VOC), the hallmark symptom of SCD. VOCs account for most hospitalizations (Yang et al., 1995; Yusuf et al., 2010) and are also associated with increased mortality rate (Platt et al., 1991). Crisis frequency worsens with age (Darbari et al., 2012). Lower fetal hemoglobin concentration, higher hemoglobin, or higher blood viscosity have been associated with higher frequent pain episodes (Nebor et al., 2011; Darbari et al., 2012, 2013). Nonetheless, these factors do not account for immediate transition from steady state to VOC, and the mechanisms responsible for initiating VOC remain elusive.

In anecdotal reports, SCD patients clearly state stress from daily events, cold exposure or pain can lead to the onset of VOC. Stress, cold exposure or pain are conditions known to activate autonomic nervous system (ANS), a major regulator of precapillary blood flow in the body. It has been suggested that ANS dysregulation of peripheral blood flow can promote vaso-occlusion (Coates et al., 2018; Veluswamy et al., 2019). ANS could be modulated by pro-inflammatory states, and in turn, could exacerbate inflammatory responses creating a vicious cycle of pain (Ballas et al., 2012). Our group has employed multiple laboratory stressors, such as hypoxia (Sangkatumvong et al., 2011), cold face stimulation (Chalacheva et al., 2015), heat pain (Khaleel et al., 2017), cold pain (Veluswamy et al., 2020), and orthostatic stress (Chalacheva et al., 2019), to determine the resulting ANS responses in SCD and control subjects. These studies demonstrated the presence of significant abnormalities in the ANS responses of the subjects with SCD. Importantly, we found that the peripheral vasoconstriction in response to heat and cold stimuli was greater in SCD.

Psychological factors play an important role in SCD patients’ quality of life. Higher stress, along with mood, has been associated with increases in self-reported SCD pain frequency and pain intensity (Porter et al., 2000; Gil et al., 2004). In turn, increases in SCD pain led to increases in stress (Gil et al., 2003). Such observations suggest the possibility that mental stress (MTS) may also play a role in the onset of VOC by exerting its impact through the ANS. In a previous study (Shah et al., 2020), we measured the cardiovascular responses of SCD and healthy subjects to MTS tasks that included N-back, Stroop, and anticipated pain tests. However, in contrast to physical autonomic stressors, such as heat pain and cold pain, where SCD subjects showed exaggerated vasoconstriction responses (Khaleel et al., 2017; Veluswamy et al., 2020), the responses of the SCD subjects to MTS were not different from their control counterparts. However, there is substantial variability in the magnitude of the responses within the SCD and control subjects. We sought to address this variability by analyzing the vasoconstriction responses of SCD and controls to MTS in the context of a more detailed and quantitative characterization of the underlying autonomic physiology. In particular, we incorporated previously unreported measurements of beat-to-beat blood pressure to estimate baseline cardiac and vascular baroreflex activity, and determined how these indices of resting autonomic function may have influenced the subsequent vasoconstriction responses to MTS.

## Materials and Methods

### Experimental Protocol

The experiments from which the present data were derived have been reported previously (Shah et al., 2020). Briefly, after 5-min of baseline, 20 SCD and 16 non-SCD subjects participated in the MTS protocol. There were two cognitive tasks (N-back and Stroop) presented in a random order and one pain anticipation task (PA) presented as the last task. The N-back task consisted of 12 sequences of alphabetic letters. The subjects responded when a letter was repeated from *n* steps (*n* = 0, 1, 2, 3) earlier in a sequence. One sequence lasted 42.5 s. There was a 25-s break between two adjacent sequences. The total duration of the N-back task was 13 min. The Stroop task consisted of 12 sequences of color words (e.g., “red,” “yellow,” and “black”). The subjects continuously responded by identifying the font color of the color word, not the written name of the word. One Stroop sequence lasted between 54 and 72 s. There was a 20-s break between two adjacent sequences. The total duration of the Stroop-task was about 15 min. The PA task was a brief instruction appeared on the screen, informing the subjects that they were about to receive the maximum pain in 1 min. However, no physical pain was given.

### Measurements and Data Processing

Measurements were carried out in a quiet, dimmed light, temperature-controlled room (27 ± 0.5°C). The subjects rested comfortably at an approximately 45° angle on a cushioned chair with arm and leg supports. The photoplethysmogram (PPG) was obtained from the thumb of the subjects’ left hand (Nonin Medical Inc., United States). Continuous blood pressure was measured from the left middle finger using a noninvasive finger cuff (Nexfin; BMEYE, Amsterdam, Netherlands). Other physical measurements include laser Doppler flowmetry (Perimed, Sweden), electrocardiogram (ECG), respiration (zRip DuraBelt, Philips) were also recorded. All measurements were acquired synchronously through Biopac MP150 data acquisition system (Biopac, United States) at 250 Hz.

Beat-to-beat values were detected with respect to the R peaks on the ECG. First, R-peaks were detected from ECG recording using our own adaptive thresholding algorithm. The R-peak was the maximum point which exceeded the 90th percentile value of the ECG recording within a 1.5-s window. The process repeated until the end of ECG recording. The identified R-peaks were used to define the beginning and the end of each cardiac cycle and were used for subsequent beat-to-beat value extraction in other signals. R–R interval (RRI) was defined as the time between two consecutive R-peaks. Systolic and diastolic blood pressure (SBP and DBP) were the peak and nadir of the blood pressure pulse during the cardiac cycle. The pulse amplitude of the PPG waveform (PPGa), which reflects changes in pulsatile finger blood volume, was measured from the difference between the peak and nadir of the PPG waveform within the cardiac cycle. We used PPGa as a surrogate measure of peripheral vascular conductance, thus assuming that decreases in PPGa reflect vasoconstriction and increases reflect vasodilation (Alian and Shelley, 2014). It has been shown that increases and decreases in PPGa generally coincide with cutaneous blood flow, measured using laser Doppler flowmetry (Rauh et al., 2003; Khoo and Chalacheva, 2019), and that these changes take place primarily as a result of peripheral vasodilation or vasoconstriction. However, it is also known that a fraction of these changes in PPGa may be due to changes in pulse pressure and/or local arterial distensibility (Khoo and Chalacheva, 2019). Since the PPG is a relative measurement, PPGa was normalized to its own 95th percentile value of its full study recording and expressed in normalized units (nu).

### Quantification of Vasoconstriction

Vasoconstriction response, ΔPPGa, to each of the tasks was defined as the difference between mean PPGa during baseline and the mean PPGa during each task. Positive values of ΔPPGa therefore represented vasoconstriction, while negative values reflected vasodilation.

### Characterization of Baseline Autonomic Function

Univariate indices of baseline autonomic function in each subject were assessed using the averages of the beat-to-beat values of RRI, SBP, DBP, and PPGa along with heart rate variability and blood pressure variability measures. To assess the variability measures, the beat-to-beat signals of RRI and SBP were initially converted into uniformly sampled time series, with the sampling interval of 0.5 s using an interpolation and resampling algorithm (Berger et al., 1986). Then, the mean and the very low frequency trend (0–0.01 Hz) were subtracted from the time series. The spectral density of RRI and SBP variability was computed using autoregressive modeling. The area of the power spectrum for RRI variability within the high-frequency band (HF: 0.15–0.4 Hz) was computed in order to obtain HFP_*RRI*_ (HF power of RRI variability), a well-accepted metric of vagal modulation of heart rate. The ratio of low-frequency (LF: 0.04–0.15 Hz) to high frequency spectral power (low-to-high ratio, LHR) was used as a broad index of “sympathovagal balance” (Task Force of the European Society of Cardiology and the North American Society of Pacing and Electrophysiology, 1996). The area of the power spectrum of SBP variability within the LF band was computed to obtain LFP_*SBP*_ (LF power of SBP variability). LFP_*SBP*_ was taken to indicate sympathetic modulation and intrinsic vasomotor rhythmicity of blood pressure (Ryan et al., 2011; Pagani et al., 2012). The cardiac and vascular baroreflex sensitivities were also estimated based on the spontaneous fluctuations in the cardiovascular variables during the baseline period using two approaches.

#### Baroreflex Sensitivities Using the “Sequence” Technique

We used the “sequence” method (Parati et al., 2000) to estimate the cardiac baroreflex sensitivity (BRS_*c*_), which reflects predominantly vagal control of the heart. In this approach, we first identified the sequences of beat-to-beat SBP and RRI that changed together in the same direction, either increasing or decreasing, for at least 3 consecutive beats. The slope of the regression line between SBP and RRI was taken to represent an estimate of BRS_*c*_ over the duration of that sequence. The change in SBP or RRI from the current beat to the next beat was required to be greater than a minimum threshold in order to qualify as an increasing or decreasing sequence (Persson et al., 2001). RRI was allowed to lag SBP by 0–5 beats (Porta et al., 2018). For each individual baseline, the optimal lag was chosen as the value that gave the maximum cross-correlation between RRI and SBP signals (closer to +1). The lag was chosen before the sequences were identified. Only the sequences with the goodness of fit (*R*^2^) of the regression line equal to or greater than 0.9 were considered in the analysis. The average BRS_*c*_ of all sequences identified in the baseline period was taken to represent BRS_*c*_ for that subject.

The vascular baroreflex sensitivity (BRS_*v*_) characterizes the factor by which increases (decreases) in DBP induce vasodilation/vasoconstriction of the peripheral vasculature. Previous studies have employed various measurements to quantify the effector variable for this reflex, including muscle sympathetic nerve activity (MSNA; Durocher et al., 2011; Marchi et al., 2016), systemic vascular resistance (Borgers et al., 2014), and skin blood flow (SBF; Porta et al., 2018). In our case, we used the changes in PPGa as the surrogate measure of changes in peripheral vascular conductance. To estimate BRS_*v*_, we used the approach proposed by Porta et al. (2018) which was based on the sequence technique used for estimating BRS_*c*_. In this case, sequences of consecutive increases (decreases) in beat-to-beat DBP values were related to corresponding subsequent sequences of parallel increases (decreases) in beat-to-beat PPGa. For each sequence, the BRS_*v*_ was defined as the slope of the linear regression of PPGa over DBP. The sequences of DBP and PPGa values were composed of three or more consecutive beats. The total change in DBP of each identified sequence had to be least 1 mmHg. The delay for a sequence of DBP-PPGa values was allowed to range from 0 to 5 beats (Porta et al., 2018). The delay was estimated *via* cross-correlation before the sequences were identified, with the “optimal” delay corresponding to the maximum positive correlation between DBP and PPGa beat-to-beat values. The average BRS_*v*_ of all sequences identified in the baseline period was taken to represent BRS_*v*_ for that subject.

#### Baroreflex Sensitivities Using the “Spectral” Technique

Since the sequence technique may be limited to assess the baroreflex function only at the high frequency oscillation (modulated by respiration), as consistency checks on the aforementioned BRSc and BRSv estimates, we also computed the corresponding baroreflex sensitivities using the “spectral” technique, which has been used extensively for quantifying BRS_*c*_ (Parati et al., 2000). The beat-to-beat signals of RRI, SBP, DBP, and PPGa were first converted into uniformly sampled time series, with 0.5 s as the interval between samples, using an interpolation and resampling algorithm (Berger et al., 1986). Subsequently, the mean and very low frequency trend (0–0.01 Hz) were subtracted, following which the power spectral density of each signal was computed using autoregressive modeling. From each power spectra, we computed the area within the LF (0.04–0.15 Hz) band in order to yield the following spectral indices: (a) LFP_*SBP*_ (LF power of SBP variability), (b) LFP_*RRI*_ (LF power of RRI variability), (c) LFP_*DBP*_ (LF power of DBP variability), and (d) LFP_*PPGa*_ (LF power of PPGa). Then, from the aforementioned spectral indices, we computed the corresponding sensitivities (conventionally referred to as “α coefficients”) for both baroreflex arms. As in previous reports (Parati et al., 2000), for the cardiac baroreflex, α_*c*_ was defined as:


$$\propto_{c}=\sqrt{\left(\frac{LFP_{RRI}}{LFP_{SBP}}\right)}$$

Along the same lines, we defined α_*v*_ for the sympathetic vascular baroreflex as:


$$\propto_{v}=\sqrt{\left(\frac{LFP_{PPGa}}{LFP_{DBP}}\right)}$$

Both spectral indices (α_*c*_ and α_*v*_) were subsequently compared against their corresponding sequence-derived measures (BRS_*c*_ and BRS_*v*_).

### Statistical Analysis

Student’s *t*-test or Wilcoxon rank sum test (when data were not normally distributed) were used to test for the differences in the continuous parameters of subject characteristics between non-SCD and SCD groups. The comparisons of dichotomous variables of subject characteristics were tested using Pearson’s Chi-squared test. The comparisons of baseline autonomic indices between non-SCD and SCD groups were tested using Student’s *t*-test or Wilcoxon rank sum test. Pearson’s correlation analysis was used to test for the relationship between baseline BRSc and baseline BRSv. The relationship between baseline BRSv and baseline PPGa was quantified *via* Pearson’s correlation analysis. The correlation analysis was also used to test for the relationship between baseline BRSc and baseline RRI. These relationships were assessed separately for non-SCD and SCD.

The relationship between baseline BRSv and vasoconstriction were analyzed in multiple steps. First, Pearson’s correlation analysis was used to determine individual association between baseline BRSv and vasoconstriction to three different tasks. Second, we performed repeated measure analysis using a linear mixed model approach (Cnaan et al., 1997) to test for the significance of stress tasks, diagnosis and their interaction on ΔPPGa. Then, we performed another repeated measure analysis where baseline BRSv was included in the model. The regression coefficients and the group average of baseline BRSv were subsequently used to adjust for the effect of baseline BRSv on ΔPPGa. In the linear mixed model, stress tasks, diagnosis and their interaction were treated as fixed effects whereas baseline BRSv was treated as a covariate. All statistical tests were accomplished using JMP^®^ Pro 14.0.0.

## Results

### Subject Characteristics

Subject demographics are summarized in Table 1. Thirty-six subjects participated in the study. However, in contrast with the previously published study (Shah et al., 2020), we selected only 30 subjects (15 non-SCD and 15 SCD) for analysis, excluding those datasets that contained low-quality or artefactual blood pressure recordings. Two of the 30 subjects had unusable blood pressure recordings during the PA task. There was no difference in age between the two groups; although, the median age of the SCD group was slightly higher than the non-SCD group. There was no difference in state anxiety and trait anxiety scores between groups. The SCD subjects were predominantly SS patients and only two subjects were SC and Sβ^+^. The difference in hemoglobin between groups was expected. The percentage of hemoglobin S (HbS%) was considered to be zero in control subjects with sickle trait as HbS% does not contribute to sickling process in sickle trait. Seven (50%) SCD subjects were on chronic transfusion, six (36%) were being treated with hydroxyurea and two (14%) were not receiving any treatment.

**TABLE 1:** Subject characteristics and hematological parameters.

	Non-SCD (*N* = 15)	SCD (*N* = 15)	*p*-Value
Diagnosis	Healthy 11 Sickle cell trait 4	Homozygous SS 13 SC 1 Sβ+ thalassemia 1	–
Sex (M/F)	9/6	7/8	0.43
Age (years)*	15.93 (8.29)	21.61(13.05)	0.23
Hemoglobin (g/dL)*	12.8 (2.4)	9.6 (1.95)	**<0.001**
Hemoglobin S (%)	–	53.1 (7.42)	–
State anxiety	27 (12)	28 (11)	0.50
Trait anxiety*	31 (12)	30 (11)	0.90

Normally distributed data are shown as mean (SE) with p-value from Student’s t-test. Non-normally distributed data, indicated by *, are shown as median (IQR) with p-value from Wilcoxon Rank Sum test. Bolded p-values indicate significance (p < 0.05).

### Baseline Cardiovascular and Autonomic Indices

**FIGURE 1:**
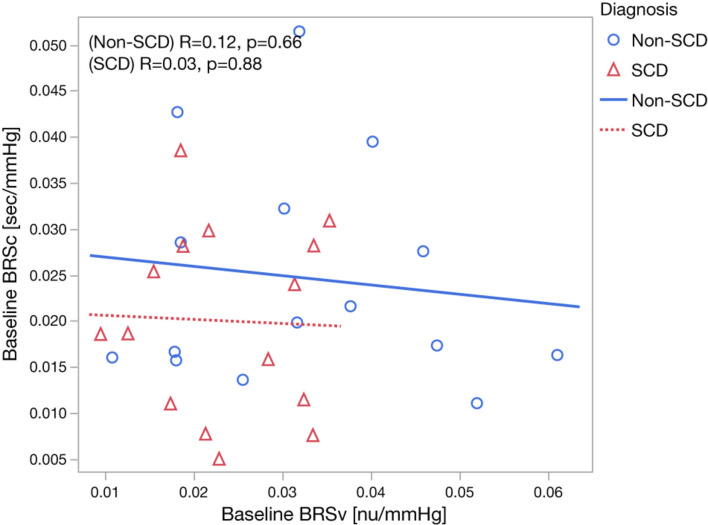
Relationship between baseline BRSv and baseline BRSc in non-SCD and SCD subjects.

The baseline cardiovascular and autonomic descriptors for all subjects studied are displayed in Tables 2, 3. The baseline mean PPGa in the SCD group was approximately 20% lower than that in the non-SCD group (*p* = 0.006). BRSv in SCD subjects was on average about 27% lower compared to non-SCD (*p* = 0.03). α_*v*_ and LFP_*SBP*_ in SCD also tended to be lower, but the group difference did not attain significance. On the other hand, BRS_*v*_ was strongly correlated with α_*v*_ (*R* = 0.83, *p* < 0.0001). Similarly, BRS_*c*_ was significantly correlated with α_*c*_ (*R* = 0.64, *p* = 0.0001). There was no significant group difference in BRS_*c*_, although there was a tendency for it to be slightly lower in SCD subjects. There was no correlation between BRSv and BRSc during the study baseline in non-SCD (*p* = 0.66) and SCD (*p* = 0.88) as shown in Figure 1.

**TABLE 2:** Baseline cardiovascular variables and total variances.

Cardiovascular/autonomic descriptor	Non-SCD (*N* = 15)	SCD (*N* = 15)	*p*-Value
RRI (sec)	0.91 (0.044)	0.84 (0.038)	0.11
SBP (mmHg)	117.16 (4.03)	115.84 (5.00)	0.42
DBP (mmHg)	71.68 (2.73)	70.58 (2.85)	0.39
PPGa (nu)	0.72 (0.033)	0.57(0.047)	**0.006**
σ^2^_RRI_* (s^2^)	0.0062 (0.0060)	0.0040 (0.0054)	0.26
σ^2^_SBP_* (mmHg^2^)	34.39 (28.95)	33.61(19.53)	0.93
σ^2^_DBP_* (mmHg^2^)	22.43 (18.43)	21.62 (18.97)	0.90
σ^2^_PPGa_* (nu^2^)	0.0695 (0.0414)	0.0337 (0.0245)	0.006

σ^2^ = variance; normally distributed data are shown as mean (SE) with p-value from Student’s t-test. Non-normally distributed data, indicated by *, are shown as median (IQR) with p-value from Wilcoxon Rank Sum test. Bolded p-values indicate significance (p < 0.05).

**TABLE 3:** Baseline autonomic descriptors.

Autonomic descriptor	Non-SCD (*N* = 15)	SCD (*N* = 15)	*p*-Value
HFP_RRI_* (s^2^)	0.00093 (0.002)	0.00089 (0.001)	0.44
LHR_RRI_*	0.88 (0.59)	1.01 (0.72)	0.44
LFP_SBP__*_ (mmHg^2^)	9.50 (10.48)	7.46 (7.23)	0.88
BRS_*c*_ (s mmHg^–1^)	0.0246 (0.0031)	0.0200 (0.0026)	0.13
BRS_*v*_ (nu mmHg^–1^)	0.0325 (0.0038)	0.0236 (0.0022)	**0.03**
α_*c*_ (s mmHg^–1^)	0.0119 (0.0015)	0.0096 (0.0010)	0.11
α_*v*_ (au mmHg^–1^)	0.0328 (0.0032)	0.0273 (0.0021)	0.08

Normally distributed data are shown as mean (SE) with p-value from Student’s t-test. Non-normally distributed data, indicated by *, are shown as median (IQR) with p-value from Wilcoxon Rank Sum test. Bolded p-values indicate significance (p < 0.05).

**FIGURE 2:**
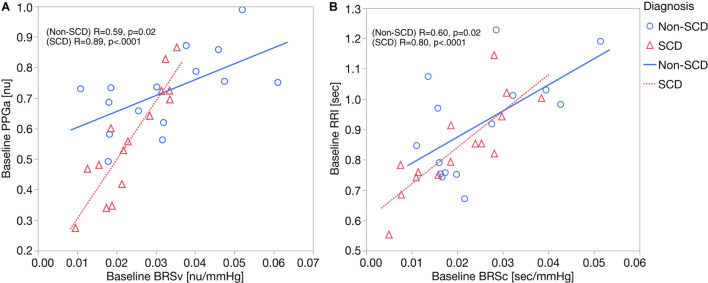
**(A)** Correlation analyses between baseline BRSv and ΔPPGa: the relationship between baseline BRSv and ΔPPGa is stronger in SCD subjects (*p* = 0.0005). **(B)** Correlation analyses between baseline BRSc and ΔRRI. No interaction was observed between baseline BRSc and diagnosis.

As displayed in Figure 2A, we found moderate-to-strong correlations between baseline mean PPGa and BRS_*v*_ in non-SCD subjects (*R* = 0.59, *p* = 0.02) and SCD subjects (*R* = 0.89, *p* < 0.0001). In the SCD group, the slope between baseline mean PPGa and BRSv was 3.7 times higher than that of the non-SCD group (*p* = 0.0005). Given that a smaller mean PPGa represents a more vasoconstricted baseline state, the lower BRSv would indicate a state of higher sympathetically mediated vascular tone.

Parallel to this finding, baseline RRI was strongly correlated to BRS_*c*_ in SCD subjects (*R* = 0.82, *p* = 0.0002) but only moderately correlated in non-SCD subjects (*R* = 0.60, *p* = 0.02) (Figure 2B). However, the slopes between baseline mean RRI and BRSc were not different between two groups. BRSc was also strongly correlated with HFP_*RRI*_ in both controls (*R* = 0.94, *p* < 0.0001) and SCD subjects (*R* = 0.82, *p* = 0.0003). The association of BRS_*c*_ with mean RRI and HFP_*RRI*_ suggests that BRSc provides a representative measure of vagal tone. We did not find correlations between the LFP_*RRI*_/HFP_*RRI*_ ratio and any of the baroreflex indices.

### Correlation Analysis Between Baseline Autonomic Indices and the Magnitude of Vasoconstriction

**FIGURE 3:**
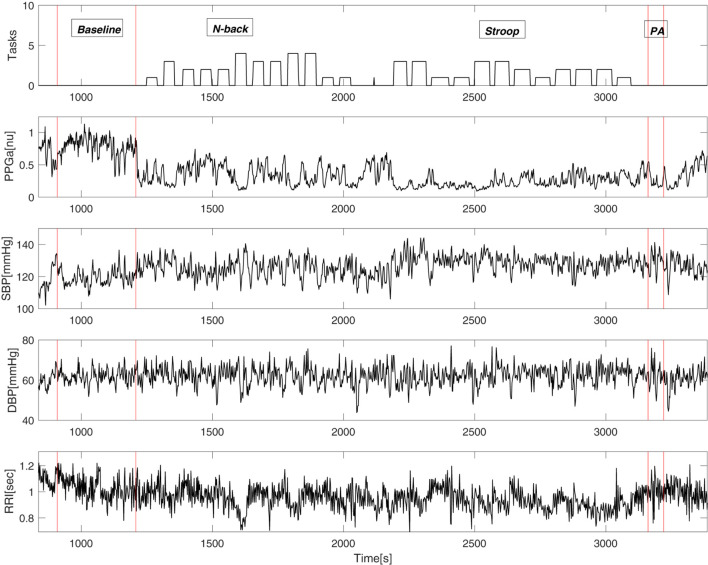
An example of cardiovascular variables during the mental stress protocol of a single subject. “Tasks” (top panel) displays the output of the E-prime software where the height of the bars represents the difficulty of the task.

Figure 3 shows an example of vasoconstriction and changes in other cardiovascular variables to MTS in one SCD subject. Once the first task began, there was a significant drop in the mean PPGa, indicating vasoconstriction. There were small increases in blood pressure and heart rate (or equivalently decreases in RRI).

**FIGURE 4:**
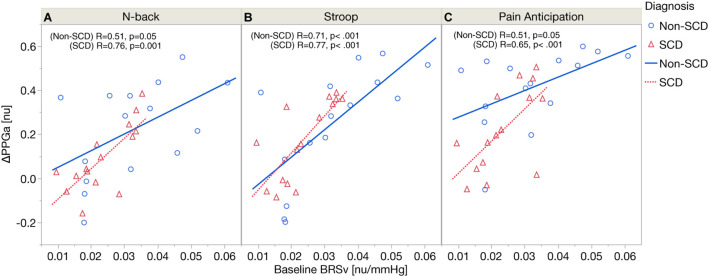
Correlation analyses between baseline BRSv and ΔPPGa in SCD and non-SCD during N-back **(A)**, Stroop **(B)**, and pain anticipation **(C)**.

Figure 4 displays the relations between baseline BRSv and ΔPPGa stratified by groups. In SCD, there was a significant correlation between baseline BRSv and ΔPPGa in N-back (*R* = 0.76, *p* = 0.0011), Stroop (*R* = 0.77, *p* = 0.0008) and the PA (*R* = 0.65, *p* = 0.0089). In non-SCD, the strongest correlation was found during Stroop (*R* = 0.71, *p* = 0.0031). The correlations between baseline BRSv and ΔPPGa were moderate during N-back (*R* = 0.51, *p* = 0.05) and PA (*R* = 0.51, *p* = 0.05). The effect of baseline BRSv on ΔPPGa was similar between SCD and non-SCD subjects.

### Effect of Mental Stress on Vasoconstriction After Adjusting for Baseline Vascular Baroreflex Sensitivity

**FIGURE 5:**
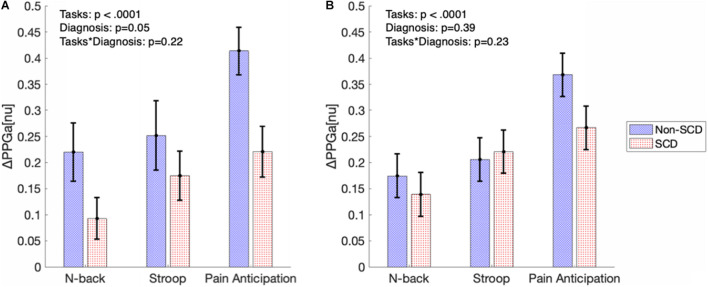
The bar graphs show magnitude of vasoconstriction (mean ± SE) in SCD and non-SCD during MTS before **(A)** and after **(B)** adjusting for baseline BRSv.

Figure 5A shows the significance of stress tasks and diagnosis on the magnitude of vasoconstriction before adjusting for baseline BRSv. Linear mixed model analysis showed that the PA task induced greater vasoconstriction than N-back (*p* < 0.0001) and Stroop (*p* = 0.007) regardless of diagnosis. In cognitive tasks (N-back and Stroop), the absolute task difficulty did not affect the magnitude of vasoconstriction. When considering all the three tasks, the magnitude of ΔPPGa tended to be lower in the SCD subjects but the diagnosis effect was not significant in the mixed model analysis (*p* = 0.05). However, when only the PA task was considered, the SCD subjects had lower responses than the controls (*p* = 0.01). Age, gender and hemoglobin were not related to the magnitude of the vasoconstriction.

Figure 5B displays the magnitudes of vasoconstriction in SCD subjects and controls, in response to the three mental tasks, after adjusting for the effect of baseline BRSv. When adjusted for baseline BRSv, there was no significant difference in ΔPPGa between SCD and non-SCD groups over the three tasks. Eliminating differences in BRSv across the subjects led on average to a reduction in the vasoconstriction response to each task in the non-SCD controls, but an increase in vasoconstriction response in the corresponding task in SCD subjects. Thus, a higher BRSv was associated with a stronger vasoconstriction response, while subjects with lower BRSv had weaker vasoconstriction responses.

## Discussion

### Role of the Baroreflexes in Modulating the Cardiovascular Responses to Mental Stress

During MTS, sympathetic outflow from the higher centers to the heart and vasculature leads to increased heart rate, peripheral vasoconstriction and consequently elevation in arterial blood pressure. These responses have been well documented in the literature (Callister et al., 1992; Hoshikawa and Yamamoto, 1997). From a theoretical perspective, the elevated blood pressure would stimulate the baroreceptors and produce compensatory changes in heart rate and cardiac contractility, as well as vascular resistance, which in turn lower blood pressure to a level consistent with the negative feedback. If this was the only factor involved, we would have expected a negative relationship between baroreflex sensitivity and the magnitude of vasoconstriction resulting from MTS, since a higher BRSv would have triggered a compensatory vasodilation to minimize the increase in blood pressure. Instead, the primary finding in this study was that the individuals with higher baseline BRSv elicited a larger vasoconstriction response to MTS in both subject groups. Thus, secondary baroreflex adjustments brought about by the primary effects of sympathetic outflow elicited by MTS were most likely **not** the predominant physiological mechanism responsible for the observed vasoconstriction responses to MTS.

An alternative hypothesis is that the strong association we observed between BRSv and the magnitude of vasoconstriction reflected an indirect, rather than direct, role of the sympathetic baroreflex. There are several pieces of evidence to support this argument. Most importantly, BRSv was strongly and positively correlated with baseline PPGa, which we have taken to represent peripheral vascular conductance. For example, subjects with low baseline BRSv tended to have low baseline PPGa, meaning that these subjects were already vasoconstricted at baseline prior to the application of MTS. As such, the magnitude of vasoconstriction (measured by the reduction in PPGa from baseline) for the same increase in sympathetic neural drive would likely be smaller due to a smaller reserve for further vasoconstriction, compared to those cases where the baseline PPGa is larger. Therefore, baseline BRSv served as a marker for the pre-MTS level of sympathetic vascular tone: the lower the BRSv, the higher the baseline vascular tone.

### Reduced Baseline Vascular Baroreflex Sensitivity in Sickle Cell Disease

The second important finding in this study was that the SCD subjects, on average, had lower baseline BRS_*v*_ than controls. As well, the SCD subjects had lower baseline PPGa (Table 2 and Figure 2A). Applying the same reasoning as had been discussed in the aforementioned section, we can only conclude that the SCD subjects had higher sympathetic vascular tone at baseline. In humans, depressed BRS_*c*_ has been reported in other cardiovascular diseases with high sympathetic tone, such as congestive heart failure (Osterziel et al., 1995). It is also well-documented that sympathetic baroreflex sensitivity decreases in response to acute cardiovascular stressors such as exercise or MTS (Durocher et al., 2011; Dampney, 2017).

The possible causes for higher sympathetic tone in SCD subjects remain unclear. Our previous work suggested that anxiety could be a factor (Shah et al., 2020); however, we did not find a significant relation between anxiety score and baseline BRSv in this work. The alteration in baseline BRSv could be due to chronic anemia (Martins et al., 2012), but we found no significant correlation between hemoglobin and baseline BRSv. Consistent with the literature, the majority of SCD subjects tend to have higher sympathetic tone, making them more vasoconstricted at baseline than the non-SCD controls. Despite a lower baseline BRSv in the SCD group, the slope between baseline BRSv and the magnitude of vasoconstriction to MTS was not different from non-SCD subjects. Therefore, the association between baseline BRSv and vasoconstriction was likely not altered by having SCD.

### Variability of Vasoconstriction Responses Resulting From Differences in Source of Autonomic Stimulation

While we found that interindividual variability of BRSv contributes to the variability in vasoconstriction responses across individuals, adjusting for differences in BRSv actually further reduced the inter-group differences in MTS-induced vasoconstriction responses (Figure 4B). This stands in contrast with the enhanced vasoconstriction responses found in SCD subjects during thermal pain stimuli (Khaleel et al., 2017; Veluswamy et al., 2020). Since there is increasing evidence that dysautonomia in SCD is associated with autonomic hyperresponsiveness, one would have expected the SCD subjects with high sympathetic tone to have displayed stronger vasoconstriction responses to MTS. This contradiction may be related to the different stressors used to elicit autonomic-mediated responses. The autonomic responses to MTS are driven predominantly by sources that have a central origin, unlike the corresponding responses to other types of stimulation that are applied peripherally (Carter and Goldstein, 2011; Dampney, 2015). Thermal pain elicits changes in sympathetic outflow *via* peripheral receptors (Morrison, 2001) while MTS triggers sympathetic outflow *via* central commands (Dampney, 2015). It is unknown whether autonomic hyperresponsiveness, if it does occur, occurs at the efferent pathways or afferent pathways of the ANS. In our previous study where the autonomic stimulus was head-up tilt, we also found no differences in vasoconstriction magnitude between SCD subjects and controls (Chalacheva et al., 2019). In the cardiovascular response to orthostatic stress, it is known that, aside from baroreflex-mediated adjustments, a strong determinant of the peripheral vasoconstriction is increased central sympathetic drive from the vestibular system (Carter and Ray, 2008).

### Estimation of Vascular Baroreflex Sensitivity

In this study, we employed a method for assessing BRS_*v*_ using the spontaneous fluctuations in DBP and PPGa that is analogous to the sequence technique commonly used to estimate BRS_*c*_. Porta et al. (2018) were the first to extend the sequence method for application to the vascular baroreflex; however, they used SBF, measured through laser Doppler flowmetry, in conjunction with noninvasive arterial pressure as the surrogate index of peripheral vascular conductance. Previous work (Rauh et al., 2003; Khoo and Chalacheva, 2019) has shown that changes in PPGa are correlated with the corresponding changes derived from laser Doppler flow. Just as the BRS_*c*_ estimates derived from the sequence technique have been found to correlate closely with the corresponding estimates deduced from the “spectral technique” (Parati et al., 2000; Persson et al., 2001), we found our estimates of BRSv from both methods to also be strongly correlated. However, it should be emphasized that these estimates of BRSv differ significantly from previous studies that have used MSNA to quantify sympathetic baroreflex output, since those studies do not take the vascular aspect of the entire baroreflex arc into account. The reflex response of MSNA to changes in arterial pressure may not necessarily induce a vascular response in a linear fashion. For instance, there was no correlation between MSNA burst incidence and the percentage change in leg vascular conductance in young women (Hissen et al., 2019; Robinson et al., 2019). Accordingly, a high sympathetic baroreflex gain derived using neural drive as the output may not linearly translate into a high value of BRSv, since mechanical and physical constraints in the blood vessels could result in a saturation of the transfer relation between the neural and vascular interfaces. SCD subjects have other factors like decreased nitric oxide availability and high levels of endothelin-1 making the vessels constrict more given the same neural input (Ergul et al., 2004; Akinsheye and Klings, 2010). Thus, at the highest levels of vascular tone, increasing sympathetic drive further may lead to further increases in MSNA but no additional increases in vasoconstriction, hence, resulting in the low BRSv values under these conditions. To the best of our knowledge, this is the first study ever to report measurements of BRS_*v*_ in subjects with SCD.

### Limitations of the Study

There were two main limitations in this study. First, the assessment of BRSv, which was originally proposed by Porta et al. (2018), was performed based on PPGa rather than the recordings of SBF *via* a laser Doppler flowmeter. Although SBF was acquired in all subjects, more than half of the subjects did not have a good quality signal due to noise (e.g., the signal’s pulsations synchronous with the heartbeat were not clearly distinguishable). Further, there was a large range in the magnitude of SBF when expressed in arbitrary units across the subjects. In 4–5 subjects, the amplitude of SBF was amplified (6—7 times larger than other subjects) probably due to small movements of the probe head relative to the tissue. In some subjects, the scale of SBF was suppressed. We speculated that this was due to a tight physical contact between the probe and the skin. While motion was minimal during the baseline period, we did observe that SBF was more sensitive to motion artifacts than PPGa. While the assumption that PPGa could be employed as a surrogate measure of peripheral vascular conductance (the inverse of peripheral vascular resistance) was necessary in the context of this study, further studies to compare BRSv derived from carefully measured SBF with the corresponding values derived from PPGa would be useful to determine whether the two indices of baroreflex sensitivity can be used equivalently.

Second, we did not have a good quantification of respiratory volume and were unable to incorporate this signal in our analysis. However, it was possible that the mechanical effects of respiration could accentuate the synchronization between blood pressure inputs (i.e. SBP and DBP) and the outputs (i.e., RRI and PPGa) which was not necessarily baroreflex origin. As a consequence, the BRS parameters derived in this present work might have lumped together the effect of BRS and the secondary effect of respiration. Nevertheless, in our study, the BRS estimation was only applied to the baseline section of beat-to-beat signals where respiration was most stable. Therefore, small fluctuations in respiration should not have significantly affected the estimated values of BRSc and BRSv.

## Conclusion

In this study, we examined the source of variability in the vasoconstriction responses of SCD and controls to MTS. We assessed both cardiac and vascular arms of the baroreflex in order to quantify autonomic function during the baseline period prior to application of MTS. Baseline BRSc was not different between SCD and non-SCD subjects, was not correlated with BRSv, and was not associated with the vasoconstriction responses to MTS tasks. BRSv in both groups was correlated with mean PPGa. Both baseline PPGa and BRSv were lower in SCD, suggesting that the SCD subjects were in a basal state of higher sympathetically mediated vascular tone.

After adjusting for differences in BRSv within and between groups, we found no difference in the vasoconstriction responses to all three mental tasks between SCD and non-SCD subjects. The implications of these findings are significant in SCD since vasoconstriction reduces microvascular flow and prolongs capillary transit time, thus increasing the likelihood for VOC to be triggered following exposure to stressful events.

## Data Availability Statement

The raw data supporting the conclusions of this article will be made available by the authors, without undue reservation.

## Ethics Statement

The studies involving human participants were reviewed and approved by the Children’s Hospital of Los Angeles Institutional Review Board. Written informed consent to participate in this study was provided by the participants’ legal guardian/next of kin.

## Author Contributions

PS and TC designed the study protocol. JS and RK performed the experimental device setup. PS, WT, MK, and JS ran the experiments and collected the data. WT performed data pre-processing, analyzed the data, and wrote the manuscript. WT, MCK, and PS interpreted the results. MCK, TC, PS, JS, and SV reviewed and edited the manuscript. All authors contributed to the article and approved the submitted version.

## Conflict of Interest

The authors declare that the research was conducted in the absence of any commercial or financial relationships that could be construed as a potential conflict of interest.

## Publisher’s Note

All claims expressed in this article are solely those of the authors and do not necessarily represent those of their affiliated organizations, or those of the publisher, the editors and the reviewers. Any product that may be evaluated in this article, or claim that may be made by its manufacturer, is not guaranteed or endorsed by the publisher.
